# Supplementary material for: Ropivacaine Administration Suppressed A549 Lung Adenocarcinoma Cell Proliferation and Migration via ACE2 Upregulation and Inhibition of the Wnt1 Pathway
Source: Int J Mol Sci. 2024 Aug 28;25(17):9334. doi: 10.3390/ijms25179334 (PMC11395614; doi:10.3390/ijms25179334)
Supplement: Supplementary file 1 [file ijms-25-09334-s001.zip › ijms-3129209-supplementary.pdf]

Table S1. The primer list for qRT-PCR and transfection

Table S1a. The oligo sequences for siRNA transfection

Gene name	Sense sequence	Antisense sequence	Assession number	Nucleotide number
ACE2 si1	5’-uuaaacuuguccaaaaauguc-3’	5’-cauuuuuggacaaguuuaacc-3’	NM_001371415.1	127-149
ACE2 si2	5'-uguuauaauuccaagaagcaa-3'	5’-gcuucuuggaauuauaacacc-3’		183-205
ACE2 sham	5'-atcaccctcttcgtcgcttc-3'			127-149

Table S1b. The primer sequences for RT-PCR

Gene name	Forward primer sequence	Reverse primer sequence	Assession number	Nucleotide number	R2 score	Efficiency
ACE2	5’-agtggatgtgatcttggctca-3’	5’-gaacaggtcttcggcttcg-3’	NM_001371415.1	20-151	0.889	98.870
HIF1alpha	5'-atcaccctcttcgtcgcttc-3'	5'-acgcggagaagagaaggaaa-3'	NM_001530.4	149-308	0.966	98.852
GAPDH	5'-ttggtatcgtggaaggactc-3'	5'-acagtcttctgggtggcagt-3'	NM_002046.4	677-718	0.976	97.896

Table S2. The array analysis of cancer related genes after ropivacaine administration (R) with or without siRNA transfection (si) compared with the control group (C).

Table S2a. Genes which were upregulated in the ropivacaine and the siRNA-treated group(s)

Assay	Relative changes				p value (q value)			
	C	R6	si	siR6	C vs R6	C vs si	R6 vs siR6	si vs siR6
CDC42	1.000 ± 0.268	2.308 ± 0.598	3.777 ± 1.067	2.479 ± 0.733	0.025 (0.174)	0.000 (0.002)	0.976 (0.999)	0.027 (0.179)
JUN	1.000 ± 0.293	3.255 ± 0.832	4.392 ± 0.757	3.968 ± 0.952	0.006 (0.059)	0.000 (0.002)	0.182 (0.671)	0.808 (0.999)

Table S2b. Genes which were upregulated in the siRNA-treated group(s) in array analysis

Assay	Relative changes				p value (q value)			
	C	R6	si	siR6	C vs R6	C vs si	R6 vs siR6	si vs siR6
FYN	1.000 ± 0.360	1.323 ± 0.530	6.446 ± 1.895	3.724 ± 0.907	0.956 (0.999)	0.000 (0.002)	0.006 (0.060)	0.002 (0.025)
ITGB3	1.000 ± 0.135	1.451 ± 0.295	4.469 ± 1.534	2.420 ± 0.308	0.945 (0.999)	0.000 (0.002)	0.326 (0.915)	0.008 (0.075)
TGFB1	1.000 ± 0.182	1.625 ± 0.369	2.975 ± 0.482	2.994 ± 0.455	0.126 (0.525)	0.000 (0.002)	0.000 (0.002)	0.999 (0.999)
WNT1	1.000 ± 0.282	3.581 ± 0.566	20.957 ± 3.234	6.361 ± 1.701	0.199 (0.726)	0.000 (0.002)	0.966 (0.999)	0.000 (0.002)

Table S2c. Genes which were upregulated in the ropivacaine group but downregulated in the siRNA-treated group(s) in array analysis

Assay	Relative changes				p value (q value)			
	C	R6	si	siR6	C vs R6	C vs si	R6 vs siR6	si vs siR6
ERBB2	1.000 ± 0.353	1.617 ± 0.359	0.608 ± 0.241	0.783 ± 0.387	0.024 (0.170)	0.220 (0.786)	0.002 (0.025)	0.809 (0.999)
HGF	1.000 ± 0.323	1.573 ± 0.330	0.166 ± 0.101	0.169 ± 0.059	0.003 (0.031)	0.000 (0.002)	0.000 (0.002)	0.999 (0.999)
IGF1	1.000 ± 0.362	3.201 ± 0.863	0.594 ± 0.211	0.774 ± 0.052	0.000 (0.002)	0.778 (0.999)	0.000 (0.002)	0.962 (0.999)
KIT	1.000 ± 0.193	2.111 ± 0.483	0.053 ± 0.015	0.155 ± 0.047	0.004 (0.042)	0.005 (0.052)	0.000 (0.002)	0.973 (0.999)
RAC1	1.000 ± 0.160	1.489 ± 0.236	0.529 ± 0.108	0.469 ± 0.108	0.041 (0.234)	0.006 (0.059)	0.000 (0.002)	0.999 (0.999)

Table S2d. Genes which were upregulated only in the ropivacaine and siRNA-treated groups in array analysis

Assay	Relative changes				p value (q value)			
	C	R6	si	siR6	C vs R6	C vs si	R6 vs siR6	si vs siR6
BCL2L11	1.000 ± 0.329	0.735 ± 0.160	0.981 ± 0.218	1.561 ± 0.479	0.494 (0.999)	0.999 (0.999)	0.001 (0.017)	0.025 (0.170)
MAX	1.000 ± 0.155	2.164 ± 0.430	1.848 ± 0.299	4.213 ± 0.924	0.096 (0.444)	0.506 (0.999)	0.000 (0.005)	0.000 (0.002)

Table S2e. Genes which were upregulated in the ropivacaine groups in array analysis

Assay	Relative changes				p value (q value)			
	C	R6	si	siR6	C vs R6	C vs si	R6 vs siR6	si vs siR6
FOS	1.000 ± 0.294	3.223 ± 1.213	0.869 ± 0.242	2.301 ± 0.708	0.000 (0.003)	0.027 (0.179)	0.000 (0.002)	0.014 (0.109)

Table S2f. Genes which were downregulated in the siRNA-treated group(s) in array analysis

Assay	Relative changes				p value (q value)			
	C	R6	si	siR6	C vs R6	C vs si	R6 vs siR6	si vs siR6
CCNE1	1.000 ± 0.283	0.878 ± 0.235	0.651 ± 0.127	0.387 ± 0.080	0.719 (0.999)	0.031 (0.193)	0.002 (0.025)	0.132 (0.542)
MAP3K5	1.000 ± 0.127	0.998 ± 0.182	0.391 ± 0.069	0.591 ± 0.080	0.952 (0.999)	0.004 (0.040)	0.322 (0.914)	0.338 (0.929)
SOS1	1.000 ± 0.126	0.697 ± 0.107	0.486 ± 0.056	0.610 ± 0.097	0.068 (0.337)	0.001 (0.016)	0.851 (0.999)	0.721 (0.999)

Table S2g. Genes which were downregulated in the ropivacaine group and the siRNA-treated group in array analysis

Assay	Relative changes				p value (q value)			
	C	R6	si	siR6	C vs R6	C vs si	R6 vs siR6	si vs siR6
CDH1	1.000 ± 0.526	0.239 ± 0.115	0.309 ± 0.132	0.620 ± 0.184	0.001 (0.016)	0.003 (0.035)	0.141 (0.565)	0.283 (0.862)

Table S3. The qRT-PCR results after ropivacaine administration (R) with or without siRNA transfection (si) compared with the control group (C).

Table S3a. The qRT-PCR results

Assay	Relative changes							
	C	R0.1	R1	R6	si	siR0.1	siR1	siR6
ACE2	1.000 ± 0.305	1.264 ± 0.142	1.269 ± 0.138	1.371 ± 0.274	0.736 ± 0.031	0.678 ± 0.232	0.544 ± 0.130	0.503 ± 0.064
BAX	1.000 ± 0.199	0.845 ± 0.250	0.746 ± 0.047	0.872 ± 0.338	1.000 ± 0.238	1.102 ± 0.282	1.343 ± 0.417	1.411 ± 0.168
BCL2	1.000 ± 0.150	0.888 ± 0.080	0.861 ± 0.108	1.286 ± 0.337	0.391 ± 0.045	0.386 ± 0.022	0.358 ± 0.090	0.480 ± 0.050
EGFR	1.000 ± 0.253	1.093 ± 0.439	0.793 ± 0.224	1.034 ± 0.039	1.673 ± 0.123	1.693 ± 0.586	1.662 ± 0.340	2.143 ± 0.859
HIF1alpha	1.000 ± 0.115	1.093 ± 0.439	0.793 ± 0.224	1.034 ± 0.039	1.673 ± 0.123	1.693 ± 0.586	1.662 ± 0.340	2.143 ± 0.859
WNT1	1.000 ± 0.478	0.774 ± 0.323	0.831 ± 0.276	1.111 ± 0.387	4.020 ± 0.931	3.436 ± 1.276	5.619 ± 2.016	1.521 ± 0.693

Table S3b. The comparison of PCR array and qRT-PCR results

Assay	RT-PCR				PCR array			
	C	R6	si	siR6	C	R6	si	siR6
BAX	1.000 ± 0.199	0.872 ± 0.338	1.000 ± 0.238	1.411 ± 0.168	1.000 ± 0.349	1.416 ± 0.346	0.910 ± 0.217	0.841 ± 0.290
BCL2	1.000 ± 0.150	1.286 ± 0.337	0.391 ± 0.045	0.480 ± 0.050	1.000 ± 0.271	0.763 ± 0.165	0.819 ± 0.190	0.623 ± 0.209
EGFR	1.000 ± 0.253	1.034 ± 0.039	1.673 ± 0.123	2.143 ± 0.859	1.000 ± 0.450	0.870 ± 0.328	1.861 ± 0.905	1.938 ± 0.498
WNT1	1.000 ± 0.478	1.111 ± 0.387	4.020 ± 0.931	1.521 ± 0.693	1.000 ± 0.282	3.581 ± 0.566	20.957 ± 3.234	6.361 ± 1.701

Abbreviations for above tables:
C: control; R: ropivacaine; si: siRNA transfection; ACE2: angiotensin-converting enzyme 2; HIF1alpha: hypoxia-inducible factor 1-alpha; GAPDH: glyceraldehyde 3-phosphate dehydrogenase; BAX: BCL2-associated X protein; BCL2: B-cell lymphoma 2; BCL2L11: B-cell lymphoma 2 like 11; CASP9: caspase 9; CCNE1: cyclin D1; CDC42: cell division control protein 42 homolog; CDH1: cadherin 1; EGFR: epidermal growth factor receptor; ERBB2: v-erb-b2 avian erythroblastic leukemia viral oncogene homolog 2; FOS: Fos proto-oncogene, AP-1 transcription factor subunit; FYN: fibroblast Yes related novel; HGF: hepatocyte growth factor; IGF1: insulin-like growth factor 1; ITGB3: Integrin beta 3; JUN: Jun proto-oncogene; KIT: KIT proto-oncogene receptor tyrosine kinase; MAP3K5: mitogen-activated protein kinase kinase kinase 5; MAX: MYC associated factor X; RAC1: Ras-related C3 botulinum toxin substrate 1; SOS1: SOS Ras/Rac guanine nucleotide exchange factor 1; TGFB1: transforming growth factor beta 1; WNT1: Wnt family member 1.
